# A multi agro-climatic reference dataset for irrigated and non-irrigated fields: France and Kazakhstan

**DOI:** 10.1016/j.dib.2026.113168

**Published:** 2026-08-12

**Authors:** Henri Bazzi, Nicolas Baghdadi, Saeideh Maleki, Sami Najem, Dias Biakhmetovich Rakishev, Aset Serkebaevich Baysakov, Adrien Selles

**Affiliations:** a UMR TETIS, University of Montpellier, AgroParisTech, INRAE, CIRAD, CNRS, Montpellier, France; b Ministry of Water Resources and Irrigation of the Republic of Kazakhstan, Department of Water Policy, Kazakhstan; c BRGM, Université de Montpellier, Montpellier, France, G-Eau, INRAE, CIRAD, IRD, AgroParisTech, Institut Agro, 1039 Rue de Pinville 34000, Montpellier, France

**Keywords:** Irrigation, Field-level data, Geospatial dataset, Semi-arid climate, Temperate climate

## Abstract

Accurate spatial information on irrigated area extents is essential for assessing agricultural water use and food production. While recent advances in optical and synthetic aperture radar (SAR) remote sensing have enabled cost-effective large-scale irrigation mapping through machine and deep learning approaches, these models still depend heavily on reliable in situ reference data. Major limitation in irrigation mapping thus resides in the scarcity and uneven global distribution of well-documented irrigated reference datasets. Here, we present a new geospatial reference dataset of irrigated and non-irrigated agricultural fields collected across two contrasting agro-climatic regions: France and Kazakhstan. The dataset was compiled through extensive field campaigns conducted in 2023 (France) and 2025 (France and Kazakhstan). It provides field-level labels of irrigation status, associated crop types, and irrigation practices. In addition, we provide ready-to-use auxiliary time series derived from Sentinel-1 SAR backscatter and Sentinel-2 vegetation indices to facilitate machine learning–based irrigation mapping. This dataset supports the development and validation of remote sensing–based irrigation products as well as the spatio-temporal transferability of deep learning models across regions, crop types, and climatic conditions.

Specifications Table

**Table:** 

Subject	Earth & Environmental Sciences
Specific subject area	Field-level reference labels of irrigated and non-irrigated plots with and Sentinel time series for irrigation mapping across regions and climates
Type of data	Raw
Data collection	Reference terrain data were collected by three field surveys for the three study sites in Kazakhstan and France for the years 2023 and 2025. Sentinel-1/2 time series were extracted from Google Earth Engine.
Data source location	France, Kazakhstan (Almaty)
Data accessibility	Repository name: Zenodo Data identification number: https://doi.org/10.5281/zenodo.19693246 Direct URL to data: https://zenodo.org/records/19693246
Related research article	None

## 1 Value of the Data

• This dataset addresses the scarcity and uneven global distribution of reference data for irrigated and non-irrigated fields, a key limitation for operational irrigation mapping. The dataset enables more robust research and benchmarking in irrigation mapping across temperate and semi-arid environments by providing well-documented field-level observations from two contrasting agro-climatic regions: France and Kazakhstan.
• The dataset supports the development, training, and validation of machine learning and deep learning models for irrigation mapping using both optical and SAR data. It includes ready-to-use Sentinel-1 backscatter and Sentinel-2 vegetation index time series, allowing direct integration into remote sensing workflows without additional preprocessing. This lowers technical barriers for researchers who may not have the capacity to perform large-scale preprocessing of satellite data.
• The dataset enables independent validation and intercomparison of existing regional to global irrigation products, helping quantify classification uncertainties. It also supports analysis of spatial transferability and model generalization across contrasting agro-climatic contexts, and evaluate the robustness of irrigation detection under varying crop types and climatic conditions. The geographic contrast between France and Kazakhstan allows testing of machine and deep learning models’ generalizations across temperate and semi-arid environments.
• Beyond its technical and scientific applications, this dataset contributes to the democratization of access to high-quality agricultural reference data, particularly in regions where field data collection is logistically difficult and costly. By reducing dependence on repeated field campaigns, it can help lower research costs and support more efficient data collection strategies. In addition, it has potential relevance for public policy and water resource management by providing reliable ground-truth information that can support decision-making in irrigation-dependent agricultural systems.

## 2 Background

Supporting irrigation strategies for effective irrigation management requires accurate spatial information on irrigated area extents to assess food production, water use, and impacts on surface and groundwater resources [1]. Advances in optical and radar satellite remote sensing enabled large-scale and cost-effective monitoring of irrigated areas. However, remotely sensed based irrigation mapping extensively rely on machine and deep learning (ML and DL respectively) algorithms (e.g., Random Forest and Convolutional neural networks (CNN)) combining mostly optical and SAR time series data [2,3]. Such applications have demonstrated strong performance for irrigation mapping but required extensive in situ reference data, whose limited availability remains a major challenge for producing annual irrigation maps. For advancing large-scale and operational irrigation mapping, the release of well-documented reference datasets of irrigated reference fields is of critical importance [4]. Such datasets facilitate and accelerate research on irrigation mapping by providing reliable ground truth for training and testing remote sensing–based irrigation mapping methods. To support research on irrigation mapping across contrasting agro-climatic contexts, we introduce a new reference dataset of irrigated and non-irrigated agricultural fields in two distinct regions, France and Kazakhstan. The dataset was compiled through large-scale field campaigns conducted in 2023 (France) and 2025 (France and Kazakhstan). It provides geospatial reference information on irrigated and non-irrigated fields, associated crop types, and irrigation practices, together with auxiliary Sentinel-1 backscattering and Sentinel-2–derived vegetation indices time series.

## 3 Data Description

The dataset of irrigated and rainfed agricultural fields collected across the three study sites is publicly available in the https://doi.org/10.5281/zenodo.19693246 repository. The dataset is organized by site and year, resulting in an independent dataset for each study site. The dataset for each site/year includes both primary field-collected data and secondary processed data derived from Sentinel-1 and Sentinel-2 imagery for the study areas.

For each site and year, the data is compressed into a “zip” file as:

• Dataset_Almaty_Kazakhstan_2025.zip
• Dataset_France_2023.zip
• Dataset_France_2025.zip

Each zipped file contains four components: (i) field boundaries in ESRI shapefile format, (ii) Sentinel-1 time-series data in CSV format, (iii) Sentinel-2 time-series data in CSV format, and (iv) a README file describing the metadata and data structure. The unique field identifier (ID) is common to the shapefile and both Sentinel-1 and Sentinel-2 CSV files, allowing direct linkage between the spatial and temporal datasets.

• **Field boundaries (shapefile format):**

A georeferenced shapefile containing the spatial boundaries of the collected agricultural fields. The associated attribute table includes information such as irrigation information (irrigation or non-irrigated), irrigation equipment (when available), crop type, and field surface area. Shapefiles for the French study sites are provided in the EPSG:2154 coordinate reference system, while the Kazakhstan study site shapefile is projected in EPSG:32643 (WGS 84 / UTM zone 43 N). Table 1 summarizes the field boundary dataset for the three sites.

• **Sentinel-1 (S1) time series data:**

**Table 1:** Summary of the reference agricultural field datasets available for each study site and year.

Study Site	Year	No. of fields	Irrigated	Non-irrigated	Main crop types	Irrigation methods *
France	2023	429	230	199	maize, soybean, sorghum, sunflower	Water gun, sprinkler, drip
France	2025	438	230	208	maize, soybean, sorghum, sunflower	Water gun, sprinkler, drip
Almaty, Kazakhstan	2025	959	874	85	corn, soybeans, forage, vegetables, fruit trees	Surface irrigation, drip

*Reported when available from field surveys.

A CSV file containing the time series of Sentinel-1 backscattering values for each reference field in VV and VH polarizations as well as the ratio VH-VV. Units for the three S1 time series are in dB. Orbit number and incidence angle (in degrees) of each S1 acquisition is also available. Each field is identified using a unique identifier (ID) consistent with the corresponding shapefile entity. The CSV file naming convention is Sentinel1_CountryName_YEAR.csv.

• **Sentinel-2 (S2) time series data:**

A CSV file providing time series of three vegetation indices (NDVI: Normalized Difference Vegetation Index, GI: Green Index, and NDMI: Normalized Difference Moisture Index) for each reference field. Field identifiers (ID) are consistent with those used in the shapefile. Vegetation indices are unitless. The CSV file naming convention is Sentinel2_ Vegetation_Indices_CountryName_YEAR.csv.

• **README file:**

A README document describing the metadata and data structure, including file organization, column names, and variable definitions for the shapefile and the two CSV files. Table 2 provides a description of the main attributes provided in the field boundary shapefiles and the Sentinel-1 and Sentinel-2 time-series datasets, including variable definitions, units, and data types.

**Table 2:** Metadata summary of the main attributes contained in the field boundary shapefiles and Sentinel-1 and Sentinel-2 time-series datasets, including variable descriptions, units, valid values (or categories), and missing-value codes. The unique field identifier (ID) is shared across all files to enable data integration.

Attribute	Dataset	Description	Units	Values
ID	Shapefile, S1, S2	Unique field identifier used to link all files: Shapefile, S1 CSV and S2 CSV	-	Integer
**Field Boundaries shapefile**				
Irrigation	Shapefile	Irrigation Status	-	(1) Irrigated, (0) non-irrigated
Irrig_mat		Irrigation equipment	-	Drip, water gun, etc.; NA if unavailable
Crop_Type		Crop type	-	Text
Surface_ha		Field area in hectares	ha	Numeric
**Sentinel-1 time series CSV**				
Date	S1-CSV	S1 acquisition date	-	Text
Time		S1 acquisition time	-	Text
VVf		Field scale radar backscatter in VV polarization	dB	Numeric
VHf		Field scale radar backscatter in VH polarization	dB	Numeric
VHf-VVf		Field scale radar backscatter ratio	dB	Numeric
VVg		Grid scale radar backscatter in VV polarization	dB	Numeric
VHg		Grid scale radar backscatter in VH polarization	dB	Numeric
relativeOrbitNumber_stop		Relative orbit number	-	Integer
Orbit_direction		Orbit direction		Ascending or Descending
Inc_angle		Radar incidence angle	-	Numeric
**Sentinel-2 Vegetation Indices time series CSV**				
Date	S2-CSV	S2 acquisition date	-	Text
NDVI		Normalized Difference Vegetation Index	Unitless	Numeric
GI		Green Index	Unitless	Numeric
NDMI		Normalized Difference Moisture Index	Unitless	Numeric

## 4 Experimental Design, Materials and Methods

### 4.1 Study sites

**Fig. 1:**
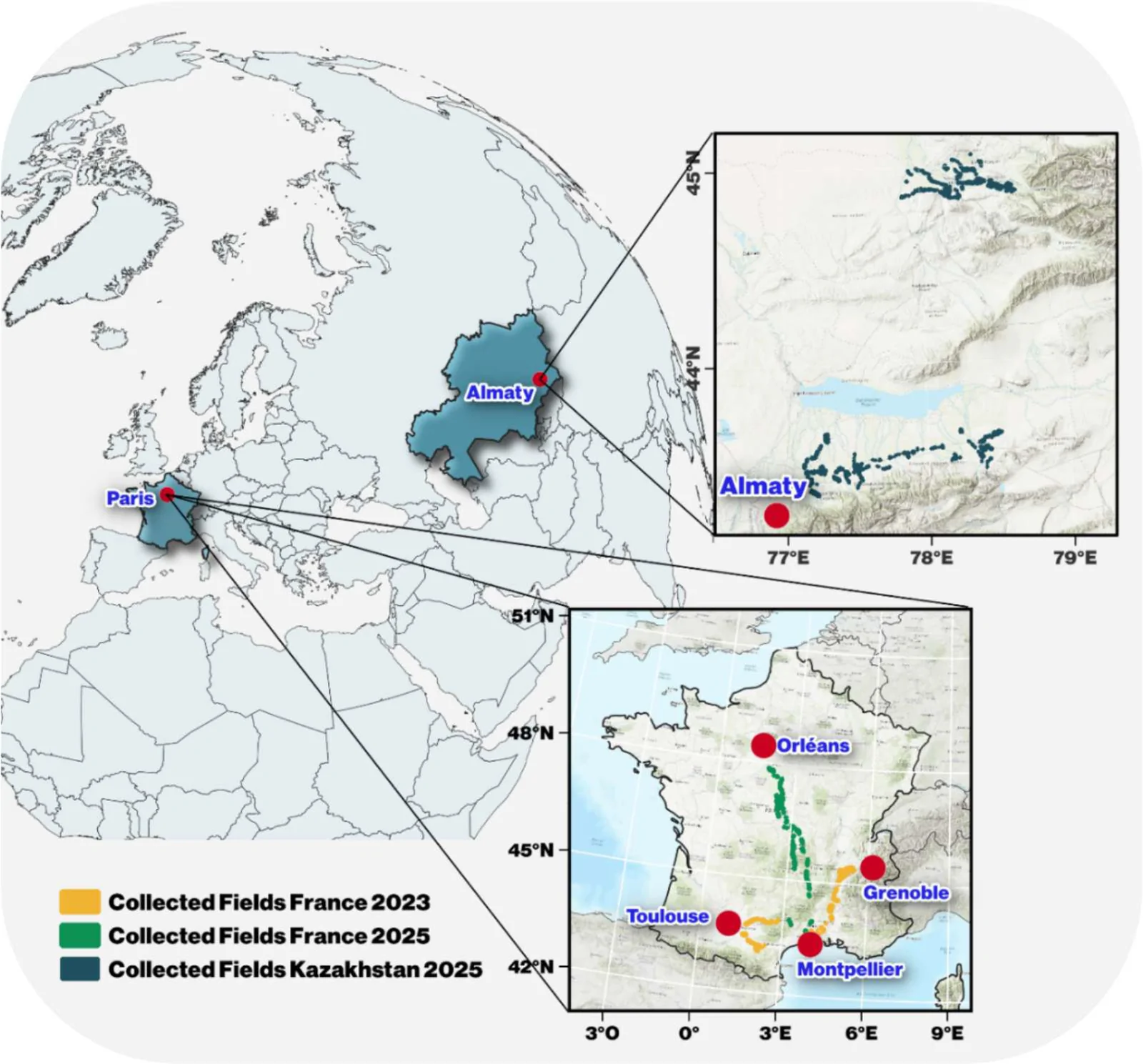
Study sites visited in France in 2023 (FR 2023) and 2025 (FR 2025), and in Kazakhstan in 2025 (KZ 2025).

Three study sites were visited for reference dataset collection. One site is in Almaty-Kazakhstan visited in 2025, and two sites are in France visited in 2023 and 2025 (Fig. 1).

#### 4.1.1 Kazakhstan, Almaty Region

The study site is located in Almaty Region in south-east Kazakhstan as shown in Fig. 1. The area has a humid continental climate strongly influenced by the Tian Shan mountains with hot dry summers and extremely cold winters. Winter is typically cold and snowy with annual total precipitation reaching about 600 mm and wettest months being April and May. Snowfall sets in December and lasts until March. Irrigation season is mainly from June to August corresponding to the warm hot summers with average temperature reaching 31 °C, and usually pushed to 35 °C with heat waves. According to farmers interviewed during field campaigns, the year 2025 was not considered a dry year.

**Fig. 2:**
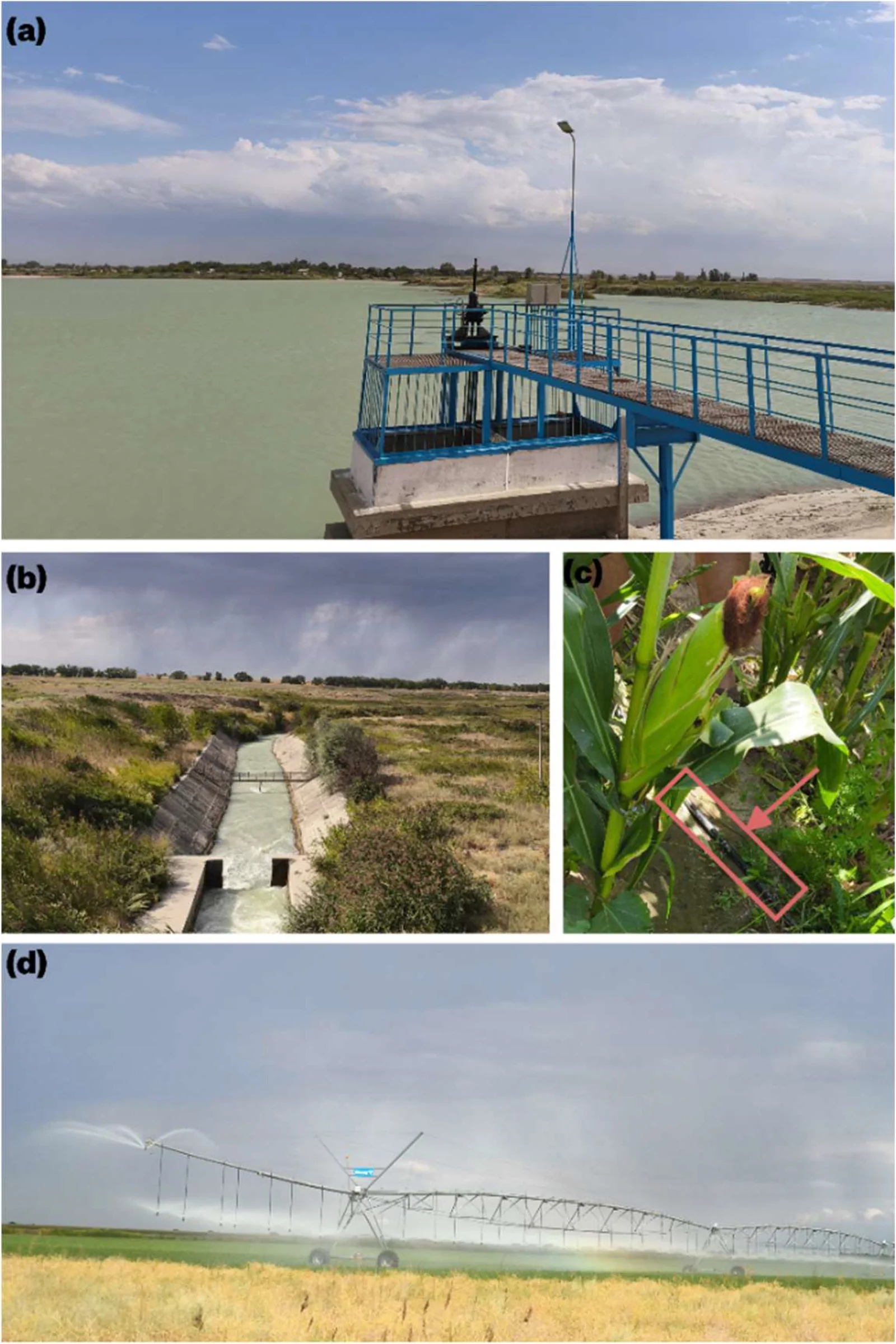
Examples of irrigation infrastructure in Almaty, Kazakhstan witnessed during the field campaign, (a) surface water lake, (b) irrigation canals for water distribution from lakes, (c) drip irrigation system for maize and (d) maize irrigated by sprinkler irrigation system.

The main sources of irrigation water in the region are surface water from lakes (Fig. 2a) distributed through water canals (Fig. 2b), then collected in local basins used for pumping water into the fields. Main irrigated summer crops are corn, soybeans, forage crops (alfalfa), horticultural crops including both vegetables crops (tomato, beetroot, etc.) and fruit trees (primarily apple).

For maize, furrow irrigation (by gravity) is principally used, with exceptionally some maize plots irrigated using hybrid irrigation system mixing between furrow and drip systems (Fig. 2c). In addition, the sprinkler system is also used for some fields (Fig. 2d). During the maize crop cycle, irrigation takes place between May and August, with 3 to 4 major irrigation events by inundation, followed in some cases by drip irrigation each 4 days each for 12 h. Depending on the meteorological conditions, farmers reported that for extremely dry years, furrow irrigation can occur once each 15 days. For soybeans, only furrow irrigation is applied with the absence of drip irrigation systems, with the same irrigation frequency as that for maize (one irrigation per month). Alfalfa is usually harvested three times between May and September and irrigated 3 to 4 times per cycle using furrow irrigation system. Fruit trees are irrigated using the drip system whereas Sunflower is rarely irrigated with some exceptions. Horticultural crops such as pepper or tomato for example, are harvested about 6 times in the summer (each 20 to 25 days) and irrigated each day. Winter wheat, sown in October and harvested in July, is usually a non-irrigated crop. However, some regions have spring wheat crops those planted in May and harvested in August. Some of these wheat fields are irrigated with usually three irrigation events between May and July.

#### 4.1.2 French sites

**Fig. 3:**
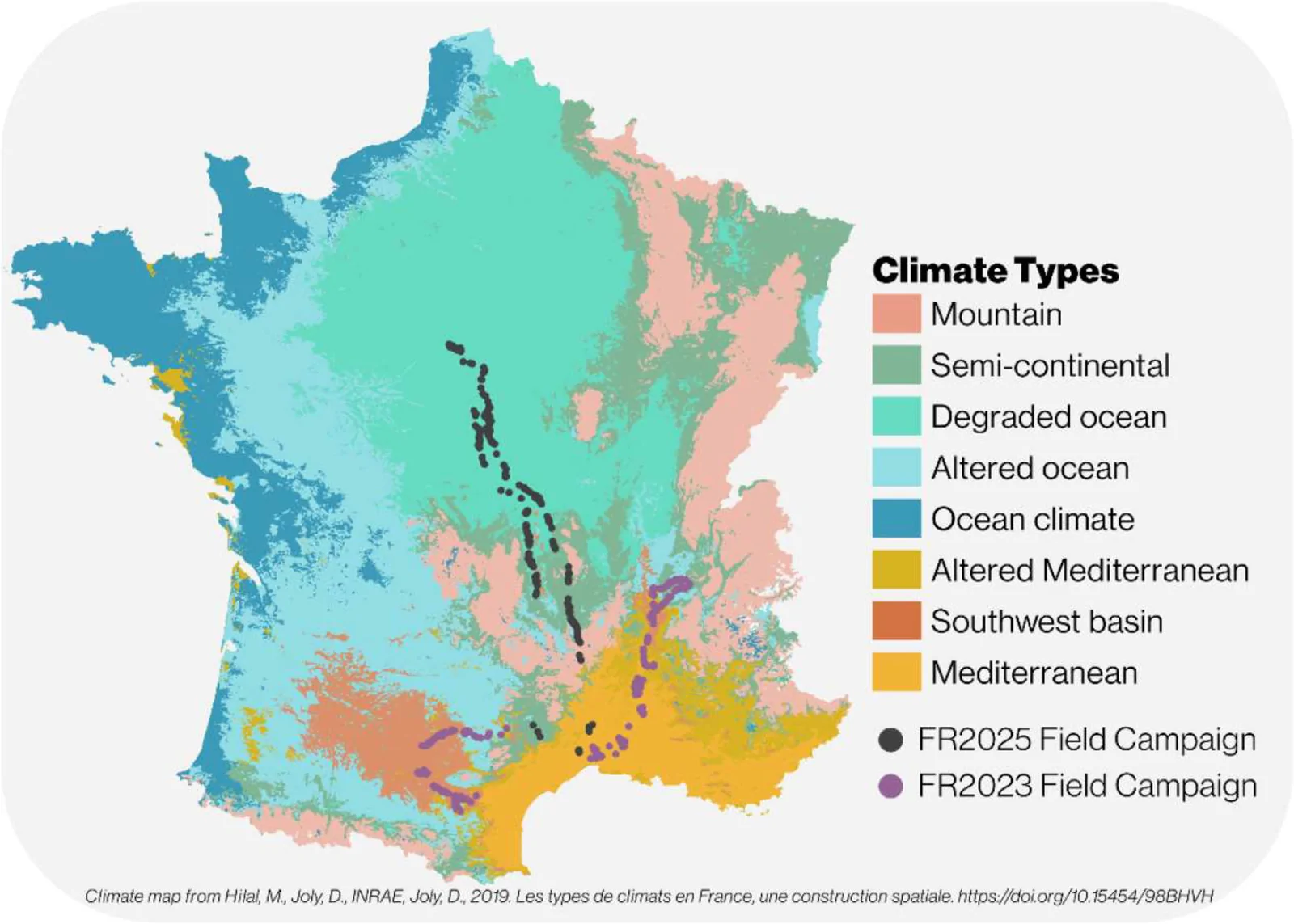
Map of French climate types and location of the collected reference fields in 2023 and 2025.

Two sites in French were visited, one in 2023 (FR2023) and the other in 2025 (FR2025) spanning different climate types [5] (Fig. 3). FR2023 site is in the south of France, starting from south east near Grenoble city (semi-continental climate), moving to the south near Montpellier city (Mediterranean) and then to south west near Toulouse city (southwest basin climate between oceanic and Mediterranean). The second study site FR2025 crosses about 500 km in France, from the south near Montpellier with a Mediterranean climate to the north passing by mountain and semi-continental climate until Orléans city with degraded oceanic climate. Major irrigated summer crops are maize, soybean and sorghum. Irrigation frequency depends heavily on the season and the climatic conditions (between Mediterranean and temperate climates) but usually varying between one to two irrigations each 10 to 20 days. Sunflower is among summer crops that are rarely irrigated. However, for both French sites, part of the summer crops is still non-irrigated, depending only on summer rainfall. Irrigation usually occurs from May to September during the summer growth cycle. Some other irrigated fields may include alfalfa and vegetable crops. Main water intake for irrigation is ground water as well as local collected water from small basins.

### 4.2 Terrain campaigns

The three terrain missions for the three study sites were organized during 2 to 5 days between July and August of the corresponding year. In Kazakhstan, local authorities accompanied us with our trip to facilitate communication with farmers, translation and collecting sufficient information. In France, no direct contact was made with local farmers. The missions were performed in three main steps:

#### 4.2.1 Pre-campaign

Prior to the terrain mission, a preliminary step consisted of obtaining proxy maps of fields limits to optimize and facilitate the detection of the fields on the terrain. For French sites, the RPG (The Graphic Parcel Register) data of the year n-1 was used to preliminary identify parcels limits, which were then validated and modified during the terrain campaign based on real observations. For the Kazakhstan site, no field parcel basemap was available. A preliminary basemap of parcels’ boundaries was obtained by performing a mean-shift segmentation using an NDVI (Normalized Difference Vegetation Index) time series of the year 2025 (from September 2024 until the nearest date before the mission) to identify approximate crop fields limits. Pre-campaign preparation also included adding a Sentinel-2 composite of RGB (natural colors) and NDVI base maps to help distinguish the visited fields. Sentinel-2 images at the nearest possible date before the mission was used to help identify clearly the crop fields. However, these preliminary fields boundaries in both Kazakhstan and France were only used to help identify the fields during the terrain campaign, while the final field boundaries were re-digitized after the campaign.

#### 4.2.2 In campaign

During the campaign, we were equipped with a tablet having the QGIS software, which allowed directly georeferencing the irrigated/non-irrigated fields with the help of the Sentinel-2 basemap and the initial plot limits prepared in the pre-campaign step. We prioritized fields with larger surface areas located near roads and accessible pathways, including agricultural tracks that could be reached by vehicle. In France, a field is noted as irrigated field if irrigation was in progress during the visit or if irrigation materials are present on the field (including pivot systems, drip systems, and continuous sprinkler systems). A field is also judged as irrigated if it contained an irrigation valve or was near an irrigation canal. Fields without any visible irrigation infrastructure were considered as non-irrigated. In addition to the irrigation information, we collected information about the crop type in the visited year, the presence of the irrigation infrastructure and the irrigation type if visible. In Kazakhstan, irrigated fields are noted based on the presence of irrigation materials (especially canals or drip irrigation system) or based on the declaration of the farmer and/or the local authorities during the survey. A field is considered non-irrigated based on the farmer’s declaration of no irrigation. Field visits to the Kazakhstan were conducted in the presence of local authorities and included informal discussions with farmers and local stakeholders to obtain contextual information on crop type, irrigation infrastructure, type and frequency. However, no personal or identifiable information was collected, recorded, or analysed, and the study did not involve human-subject research requiring ethics approval.

#### 4.2.3 Post campaign

Following the field campaign, post treatment included a detailed digitizing of each field with the help of a time series of Sentinel-2 image acquired from June to September of each year along with recent Google Earth images as basemaps. This approach ensured precise digitization of homogeneous fields, minimizing errors related to borders or mixing with neighbouring fields. Proxy maps prepared pre-campaign were not used in the post campaign digitizing. Each field is thus designated as a polygon, stored in a shapefile format with an attribute table associating the collected field information (irrigated/non-irrigated, crop type and irrigation type). A dataset for each mission is thus created: 429 fields for the FR2023 site (230 non-irrigated and 199 irrigated), 438 fields for the second French site (FR2025) with 230 non-irrigated field and 208 irrigated field and finally 959 fields in Kazakhstan in 2025 with 85 non-irrigated field and 874 irrigated field.

### 4.3 Auxiliary satellite data

To provide researchers with ready-to-use datasets for irrigation mapping using remote sensing, we provided, in addition to the geospatial parcels’ dataset, time series database based on Sentinel-1 SAR backscattering and Sentinel-2 optical vegetation indices. For each reference field, radar and optical time series were extracted over the period from early September of year n–1 to late October of year n.

For Sentinel-2 data, time series of three vegetation indices were calculated for each reference field: the NDVI, the GI and the NDMI (Table 3). These indices provide information on the chlorophyll status of vegetation and are widely used for irrigation mapping applications using remote sensing data [2,6,7]. All computations were performed using Google Earth Engine using the Sentinel-2 harmonized surface reflectance product of Level 2A (S2_SR_HARMONIZED), available on (https://developers.google.com/earth-engine/datasets/catalog/COPERNICUS_S2_SR_HARMONIZED, last accessed 25 June 2026). Sentinel-2 images were selected using a maximum cloud cover threshold of 50% at the tile level. Field-level statistics were then computed after applying a cloud mask derived from the Sentinel-2 Scene Classification Layer (SCL), retaining only pixels with a cloud probability lower than 1%. This additional filtering step was implemented to minimize the influence of residual cloud contamination and ensure the reliability of the extracted spectral indices.

**Table 3:** General and Sentinel-2 based equations for the produced vegetation indices.

Index	General Equation	Sentinel-2 bands
NDVI	$\frac{\rho_{\text{NIR}}-\rho_{\text{RED}}}{\rho_{\text{NIR}}+\rho_{\text{RED}}}$	$\frac{B08-B04}{B08+B04}$
GI	$\frac{\rho_{\text{NIR}}}{\rho_{\text{GREEN}}}$	$\frac{B08}{B03}$
NDMI	$\frac{\rho_{\text{NIR}}-\rho_{\text{SWIR}}}{\rho_{\text{NIR}}+\rho_{\text{SWIR}}}$	$\frac{B08-B11}{B08+B11}$

For Sentinel-1 data, a time series for each field was obtained from all available Sentinel-1 acquisitions at both ascending (afternoon) and descending (morning) orbits, processed also using the Google Earth Engine. The S1 Ground Range Detection product (S1_GRD) was used (https://developers.google.com/earth-engine/datasets/catalog/COPERNICUS_S1_GRD, last accessed on 25 June 2026). The Sentinel-1 GRD product available in Google Earth Engine consists of calibrated Sentinel-1 imagery that has undergone both radiometric and geometric (terrain) correction. For each reference field, the mean radar backscatter signal in both VV and VH polarizations, as well as the difference (VH-VV), were calculated by averaging all pixels within the parcel, including an inner buffer of 10 m (one pixel) to avoid pixels with field borders contribution. VV and VH backscattering values are provided in decibels (dB). Additional metadata, including the orbit number and the incidence angle of each S1 acquisition used, were also extracted at each field. All orbital passes were considered. For each plot and acquisition date, the database provides VV and VH backscatter values together with the corresponding orbit number and local incidence angle.

In addition to the field scale S1-VV and S1-VH time series, we further computed for each field, the VVg (VV at grid scale) and VHg (VH at grid scale) indices, representing a grid scale VV and VH time series for each field. For each field, a VVg and VHg value was derived by averaging all S1 pixels for all agricultural parcels with low vegetation cover (NDVI < 0.4) located within a 5 km × 5 km grid centered on the reference parcel. Indeed, studies have shown that the use of the buffered 5 km S1 time series helps improving the detection of the irrigation events and irrigated plots assuming that the SAR signal at this scale for bare soil fields is primarily driven by soil moisture changes due to rainfall, which may further help distinguish changes in soil moisture due to irrigation or rainfall [8,9]. For France, the RPG data was used to extract agricultural fields limits surrounding the reference field for the 5 km scale average (ignoring thus urban forests and other non-agricultural classes) whereas in Kazakhstan, the fields boundaries derived from the pre-campaign segmentation (section 4.2.1) were used.

**Fig. 4:**
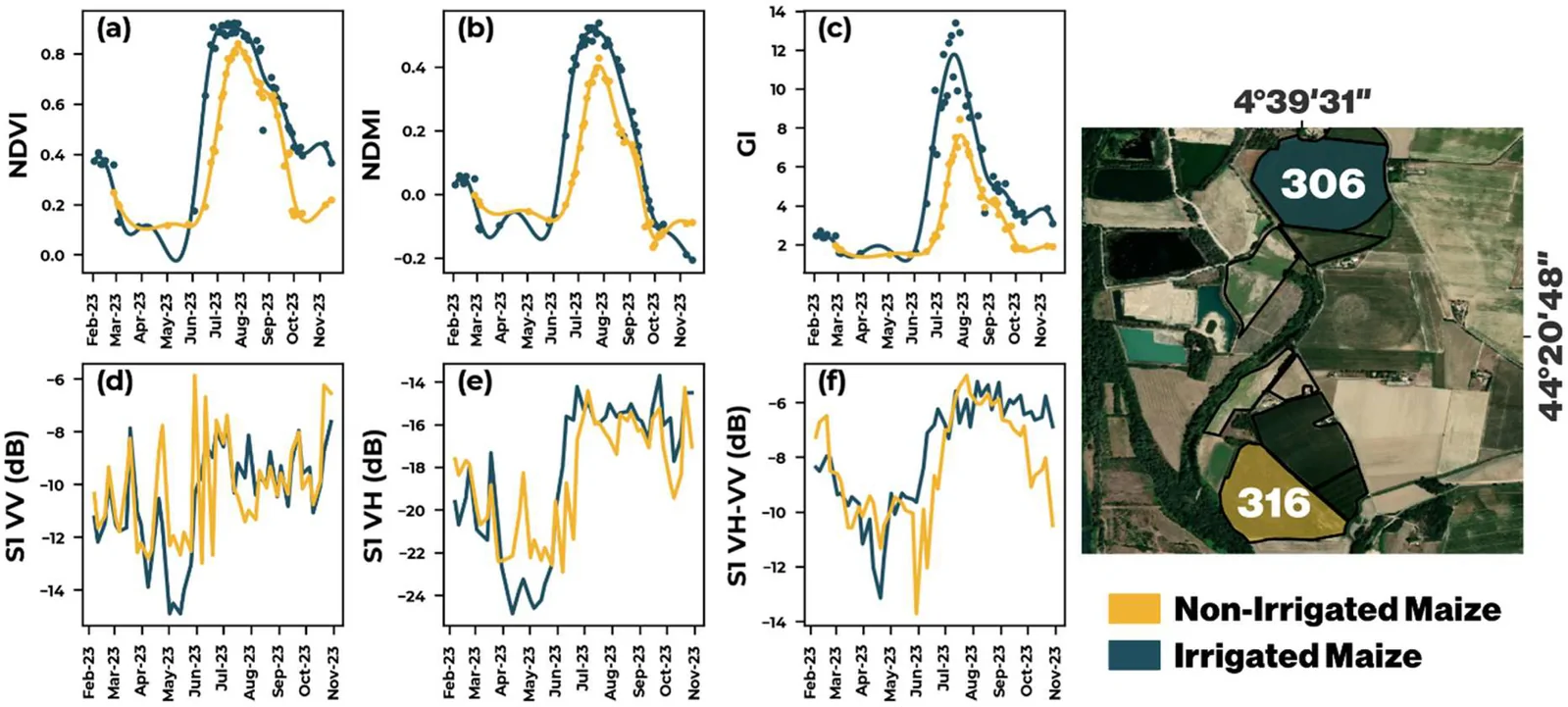
Example of the time series of (a) NDVI, (b) NDMI, (c) GI, (d) S1 VV, (e) S1 VH and (f) S1 VH-VV provided in the dataset for one irrigated (blue line) and one non-irrigated (yellow line) maize fields from the FR2023 terrain mission (ID 306 and ID 316 respectively in the database). For optical vegetation indices, dots show the raw data provided in the dataset while the continuous smoothed line represents smoothed curve using a Whittaker smoother, not provided in the dataset.

Fig. 4 illustrates example time series of NDVI, NDMI, GI, S1-VV, S1-VH, and the S1 VH-VV ratio for two maize plots, one irrigated and one non-irrigated, in France during the FR2023 campaign. For the optical vegetation indices, dots represent the raw observations from the available dataset, while the continuous lines correspond to Whittaker-smoothed curves [10] used to improve visualization. The three vegetation indices, particularly GI, clearly highlight contrasting responses between irrigated and non-irrigated maize, with the irrigated plot exhibiting higher peak values and a longer growing season. In contrast, S1-VV and S1-VH alone do not show clear visual difference between irrigated and non-irrigated maize.

### 4.4 Quality control

The dataset presented in this study presents primary data comprising field collections and associated crop type and irrigation data and secondary data including satellite time series derived from Sentinel-1 and Sentinel-2 observations. Concerning the primary data, field boundaries were manually delineated using high-resolution imagery from Google Earth, complemented by Sentinel-2 RGB composites time series acquired from June to September of each year. The delineation was performed to ensure both crop homogeneity within each field based on Sentinel-2 imagery and accurate boundary definition using Google Earth reference images. Users may apply an internal buffer to the provided field polygons to further reduce potential contamination from neighbouring land covers in pixel-based analyses. Alternatively, field boundaries may be refined or re-digitized using higher-resolution imagery when available for specific applications requiring greater spatial accuracy.

For S2 data time series, the applied cloud masking as well as the cloud threshold on the whole tile, may lead to missing data and irregular time intervals from one field to another. However, these missing observations and irregular temporal sampling were not specifically treated in the distributed CSV files. The dataset provides the original spectral indices values after cloud filtering, without any gap filling, temporal interpolation, or smoothing. This choice was made to preserve the raw observations and maximize the flexibility of the dataset, allowing users to apply their preferred methods for interpolation, filtering, or time-series smoothing according to their specific application and requirements.

Regarding the S1 time series, acquisitions from multiple orbits passing may lead to variable backscattering values depending on the incidence angle. Through literature, several methods were proposed to normalize SAR backscattering to a reference incidence angle and remove the SAR-incidence dependency in SAR time series [11,12]. In the published dataset here, no specific incidence-angle normalization was applied for the time series, allowing users to implement their preferred correction or harmonization approach rather than being constrained to a single predefined normalization method. However, incidence angle values and relative orbit number are available in the time series data for each field and at each acquisition date, which helps users apply their own incidence angle normalization.

## Limitations

Some limitations of the dataset should be considered when developing applications based on these data. First, the S1 and S2 information is provided as field-level statistics rather than pixel-level observations. While field-scale analysis is widely adopted in irrigation mapping and agricultural monitoring studies, some applications may require preserving within-field spatial variability. Some researchers prefer also performing pixel-based classifications rather than field scale (object based) classification. In such cases, users are encouraged to re-extract pixel-level satellite observations from the original S1 and S2 imagery using the provided field boundaries. Additionally, although field boundaries were carefully delineated using high-resolution imagery and field observations, small geometric inaccuracies may remain, particularly along field edges.

Another limitation is related to the geographic and sampling design of the dataset. Although the dataset covers multiple agricultural regions, crop types, irrigation practices, and environmental conditions, the sampled fields remain sparsely distributed within very large agricultural landscapes. This characteristic may limit the effectiveness of methods that explicitly exploit spatial context, such as certain deep learning architectures relying on neighborhood information or landscape-scale spatial patterns. The dataset was primarily designed for field-level classification, irrigation mapping, and time-series analysis rather than for applications requiring dense spatial coverage. In Kazakhstan, the dataset reflects the current agricultural conditions in the study area, where irrigated summer crops predominate and gravitational irrigation is the prevailing practice, resulting in relatively fewer non-irrigated field samples. This imbalance might influence the performance of machine learning models that are sensitive to class size and sample distribution.

Finally, the dataset relies on visual field visits and delineation during specific observation periods. As agricultural practices, irrigation methods, and field boundaries may evolve over time, users should consider the temporal representativeness of the reference information when applying the dataset to long-term analyses or transferring models to other regions and years.

## CRediT Author Statement

**Henri Bazzi:** Conceptualization, Methodology, Data curation, Formal analysis, Investigation, Writing–Original draft preparation. **Nicolas Baghdadi:** Conceptualization, Methodology, Formal analysis, Investigation, Visualization, Writing–Reviewing and Editing, Supervision. **Saeideh Maleki:** Data curation, Visualization. **Sami Najem:** Methodology, Data curation, Visualization, Writing–Reviewing and Editing. **Dias Biakhmetovich Rakishev:** Investigation, Writing–Reviewing and Editing. **Aset Serkebaevich Baysakov:** Investigation, Writing–Reviewing and Editing. **Adrien Selles:** Writing–Reviewing and Editing, Supervision.

## Ethics Statement

Authors have read and follow the ethical requirements for publication in Data in Brief and confirming that the current work does not involve human subjects, animal experiments, or any data collected from social media platforms.

## Data Availability

zenodoA multi agro-climatic reference dataset for irrigated and non-irrigated fields: France and Kazakhstan (Original data)
